# Dispersion engineering by rotational symmetry breaking in an optical microcavity

**DOI:** 10.1038/s41377-025-02169-2

**Published:** 2026-01-22

**Authors:** Jian-Zheng Ren, Li-Jie Li, Rui-Qi Zhang, Zhi-Yan Wang, Qi-Tao Cao, Yun-Feng Xiao

**Affiliations:** 1 State Key Laboratory for Mesoscopic Physics, Frontiers Science Center for Nano-optoelectronics, New Cornerstone Science Labotatory, School of Physics, Peking University, Beijing, 100871 China; 2 Collaborative Innovation Center of Extreme Optics, Shanxi University, Taiyuan, 030006 China

**Keywords:** Microresonators, Nonlinear optics

## Abstract

Dispersion engineering is pivotal for nonlinear optics, yet it often faces challenges posed by material and structural limitations. Here, we establish rotational symmetry breaking as the guiding principle for dispersion engineering in optical microcavities. Through boundary deformation, multi-branch global dispersion emerges in island modes, and local dispersion is controlled via resonance-assisted tunneling between quasi-whispering gallery modes. Enabled by the global dispersion, the optical parametric oscillation is predicted in blue-violet light spectrum with high efficiency (>55%) and large frequency separation (>180 THz). Using the local dispersion engineering, the doubly-resonant enhancement of second-harmonic generation is regulated by the resonance-assisted tunneling.

## Introduction

Optical dispersion engineering lies at the heart of modern optics, governing coherent control and the generation of light across a broad spectrum^1–5^. Especially in nonlinear photonics, dispersion engineering enables precise manipulation of phase-matching conditions, which is essential for various applications ranging from laser frequency extension^6,7^, optical parametric oscillation^8,9^ to quantum light sources^10–12^. For instance, in second-harmonic generation (SHG), phase mismatch can prevent energy transfer between interacting waves, drastically reducing conversion efficiency^13^. Dispersion engineering addresses this challenge by tuning the phase velocity of different frequency components, ensuring constructive interference across the entire nonlinear interaction length. In general, achieving the ideal dispersion profile requires balancing two primary factors: intrinsic material dispersion determined by the refractive index properties, and geometric dispersion based on the structural design of photonic devices. While intrinsic dispersion is material-specific and difficult to modify, geometric dispersion offers a critical degree of freedom for tailoring the overall dispersion landscape, overcoming material limitations and facilitating more flexible, broadband, and high-performance nonlinear interactions^14,15^.

Central to these advancements of dispersion engineering are high-quality optical microcavities^16–18^, which have emerged as versatile platforms for a variety of nonlinear processes, such as SHG^19–21^, three-/four-wave mixing^22–25^, stimulated nonlinear scattering^26–28^, and optical microcombs^29,30^. By carefully controlling the dispersion within microcavities, exotic interactions between light and different types of matter—such as phonons^31^, electrons^32,33^, or even excitons^34^—can be strongly enhanced. Nevertheless, the conventional paradigm of dispersion control in optical microcavities is usually constrained by symmetric geometries, which cannot optimally compensate for the baseline from material dispersion, thus restricting the efficient frequency conversion across wide frequency ranges.

Here, we present the dispersion properties of asymmetric resonant cavities (ARCs) and predict effective engineering for both global and local dispersion. The multi-branch global dispersion of island modes is uncovered across a broad spectral range driven by angular momentum spanning. Concurrently, local dispersion is engineered in-situ through resonance-assisted tunneling (RAT)^35^, which allows selective control of specific mode resonances. Assisted by the global and local dispersion engineering strategies, nonlinear photonic applications are enabled, including the highly efficient optical parametric oscillation (OPO) in the visible band, and the double-resonance enhanced SHG, respectively. These results highlight the immense potential of ARCs for ultra-efficient laser frequency extension and provide a blueprint for multifunctional integrated photonic devices.

## Results

**Fig. 1:**
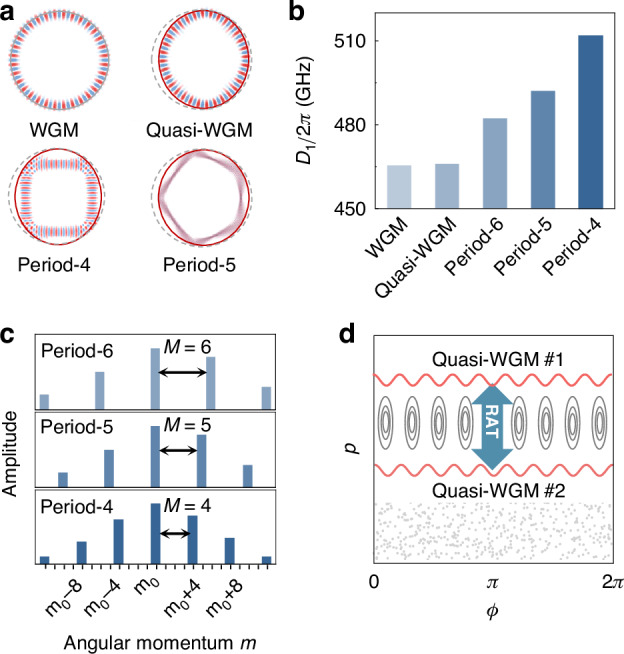
Schematic of mode fields and dispersion characteristics in ARCs. **a** Typical field distributions in real space for quasi-WGM and island modes with different periods $M$, referenced to a WGM in a circular cavity. Dashed grey lines and red solid lines represent the boundary of the circular cavity and ARC. **b** First-order dispersion coefficient ${D}_{1}$ for WGM, quasi-WGM, and island modes. **c** Angular momentum components of each island mode, where the equidistant spacing $M$ is determined by the island period. **d** Conceptual illustration of the RAT-induced coupling between two quasi-WGMs in phase space spanned by azimuthal position $\phi$ and momentum $p=\sin \chi$ of light rays

In an asymmetric microcavity, the field distribution of resonant modes is modulated by boundary deformation (Fig. 1a)^17,36–39^, giving rise to two types of long-lived modes, i.e., island modes and quasi-whispering gallery modes (quasi-WGMs). Quantitatively, the resonance frequency of the mode family can be expressed as ${\omega }_{\mu }={\omega }_{0}+{\sum }_{{\rm{i}}}{D}_{{\rm{i}}}{\mu }^{{\rm{i}}}/{\rm{i}}!$, where ${\omega }_{0}$ is the central reference frequency, $\mu$ is the mode index, ${D}_{{\rm{i}}}$ is the i-th order dispersion coefficient, and ${D}_{\mathrm{int}}={\omega }_{\mu }-{\omega }_{0}-\mu {D}_{1}$ denotes the integrated dispersion profile^29,40–42^. The deformed boundary is described in polar coordinates as

1 $$R\left(\phi \right)={R}_{0}+{R}_{0}\varepsilon \left\{\begin{array}{c}{a}_{2}{\cos }^{2}\phi +{a}_{3}{\cos }^{3}\phi \; {\cos}\phi \ge 0\\ {b}_{2}{\cos }^{2}\phi +{b}_{3}{\cos }^{3}\phi \; {\cos}\phi < 0\end{array}\right.$$

which is a typical and widely studied boundary for both rich structures in phase space and high-quality factors (see details in section I of Supplementary Information). Using the 2D finite element method, the resonance spectra are obtained at the 200 THz band, from which the first-order dispersion coefficient *D*_1_ is extracted through linear fitting. Here, the material dispersion is considered based on the cavity of Si_3_N_4_ (700 nm thickness, *R*_0_ = 50μm, *ε* = 1/3) on the SiO_2_ substrate. As shown in Fig. 1b, the first-order dispersion *D*_1_ of the island modes is larger than that of quasi-WGMs and increases with the smaller island period, which arises from the different round-trip lengths of the associated light rays in real space.

The integrated dispersion profile ${D}_{\mathrm{int}}$ of different modes can be understood through analyzing the classical trajectories of long-lived modes on the Poincaré surface of section, spanned by the azimuthal angle $\phi$ and tangential momentum $p=\sin \chi$ with $\chi$ being the incident angle (see Fig. S1 of Supplementary Information). The island modes, which exhibit polygon-like patterns without rotational symmetry, are supported by stable periodic orbits. In this case, the resonance modes are no longer eigenstates of angular momentum but a superposition of collective WGMs with multiple angular momenta^43,44^, where the equidistant spacing $M$ in the momentum space is determined by the orbit period (Fig. 1c). For quasi-WGMs supported by nearly continuous regular orbits (Fig. 1d), the counterintuitive modal coupling emerges between different azimuthal mode families, instead of the orthogonality of WGMs in a circular cavity. This coupling is constructed through the RAT process, supported by stable periodic orbits^35,45,46^, offering a flexible way of engineering local dispersion.

### Global dispersion engineering

**Fig. 2:**
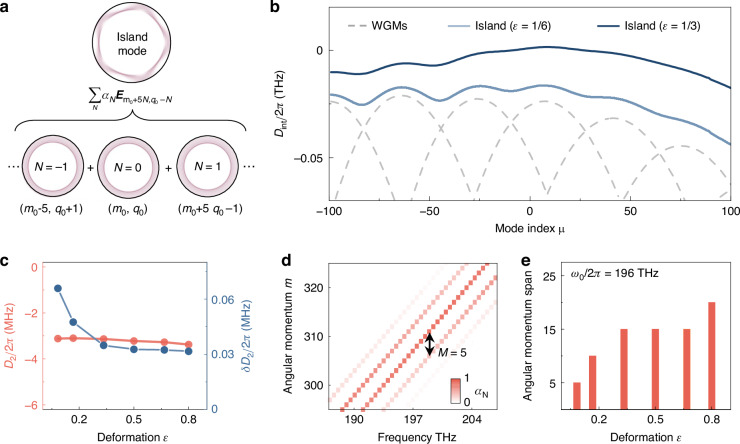
Dispersion engineering of island modes. **a** Composition of island modes ${{\boldsymbol{E}}}_{{\rm{island}}}$ as a superposition of WGMs, governed by the selection rule. **b** ${D}_{\mathrm{int}}$ for the period-5 island mode family at deformation $\varepsilon$ = 1/6 (light blue curve) and $\varepsilon$ = 1/3 (dark blue curve). Gray dashed curves represent ${D}_{\mathrm{int}}$ for different WGM families in a circular cavity. Different dispersion curves (grey dashed curves) correspond to different radial mode families. The WGMs with the same mode index $\mu$ from different mode families differ by 5 in azimuthal mode number and by -1 in radial mode number. **c** Second-order dispersion coefficient ${D}_{2}$ (red dots) and its uncertainty ${\delta D}_{2}$ (blue dots) versus deformation parameter $\varepsilon$. **d** Angular momentum composition for the period-5 island mode family at $\varepsilon$ = 1/3, showing that each mode is composed of WGMs with a momentum interval $M$ = 5. **e** Angular momentum span, defined as 10 dB bandwidth in momentum space, is presented for the period-5 island mode family versus the deformation

For the period-$M$ island modes ${{\boldsymbol{E}}}_{{\rm{island}}}$, the superposition of the nearly degenerate WGMs follows selection rules governed by the Fermi resonance condition^45^, expressed as ${{\boldsymbol{E}}}_{{\rm{island}}}={\sum }_{{\rm{N}}}{a}_{{\rm{N}}}{{\boldsymbol{E}}}_{{{\rm{m}}}_{0}+{\rm{M}}\cdot {\rm{N}},{{\rm{q}}}_{0}-{\rm{N}}}\,\left(N{\mathbb{\in }}{\mathbb{Z}}\right)$, as illustrated in Fig. 2a (see Materials and methods). Here, ${m}_{0}+M\cdot N$ and ${q}_{0}-N$ refer to the azimuthal and radial mode numbers, respectively, and ${a}_{{\rm{N}}}$ denotes the superposition coefficient. Hence, the global dispersion of the island mode family is strongly distinct from that of conventional WGMs, as shown in Fig. 2b. The integrated dispersion profiles ${D}_{\mathrm{int}}$ (light and dark blue curves) exhibit fluctuations that closely follow the envelope of ${D}_{\mathrm{int}}$ for the contributing WGMs (gray dashed curves), indicating the superposition relations in Fig. 2a. Additionally, ${D}_{\mathrm{int}}$ of island modes is much flatter compared to the global dispersion of the uncoupled WGMs, as the geometric dispersion is suppressed by the wavelength-independent light trajectories constrained along the stable periodic orbit. The second-order dispersion coefficient ${D}_{2}$/2π, extracted at approximately -3 MHz, is drastically reduced to merely 4% of its value in WGMs, unlocking the potential for broadening the spectral span of microcombs^30^. With the larger deformation coefficient *ε* (Fig. 2c), *D*_2_ preserves almost invariant, showing robustness against fabrication errors. Simultaneously, the uncertainty *δD*_2_ decreases, leading to a smoother dispersion curve as *ε* increases.

Owing to the multiple angular momentum components present in an island mode, the complete global dispersion landscape—the relationship between the resonance frequency and angular momentum—reveals a distinctive multi-branch structure, as shown Fig. 2d. The angular momentum in period-5 island mode shows an equidistant spacing $M$ = 5 and spans over 15, where the spanning range of momentum can be extended by increasing the deformation parameter $\varepsilon$ (Fig. 2e). This is because stronger cavity deformation breaks more nearly continuous regular orbits and increases the enclosed area of stable periodic orbits, thereby expanding angular momentum of the island mode by incorporating more WGM components. The dependence of the global dispersion on other boundary parameters (${a}_{i},\,{b}_{i}$) also agrees with the prediction by ray dynamics (see details in section II of Supplementary Information). Compared to the dispersion folding in a microring with varying cross-section^47^, the momentum interval of multi-branch dispersion is larger in ARCs and can be controlled by the island period, which provides wider and flexible momentum compensation.

### Local dispersion engineering

**Fig. 3:**
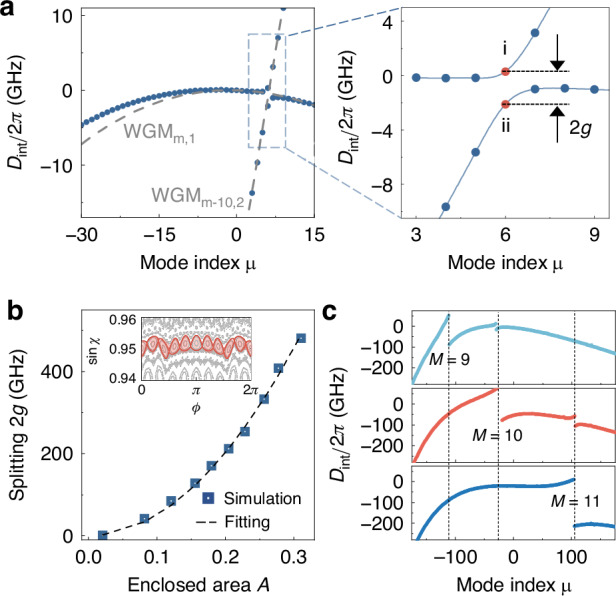
Dispersion engineering of quasi-WGMs. **a** ${D}_{\mathrm{int}}$ of the quasi-WGMs (blue dotted curves) and uncoupled WGMs (gray dashed curves). Right panel: Zoomed-in view of the avoided mode crossing region, where $g$ denotes the coupling strength, dots i and ii represent the island and scar modes. **b** Dependence of frequency splitting 2$g$ on the enclosed area $A$ of the period-10 stable periodic orbit. Inset: Phase space representation of the period-10 stable periodic orbit, where the red region denotes the enclosed area. **c** ${D}_{\mathrm{int}}$ of quasi-WGMs with selectively increasing enclosed area $A$ for period-9, 10, 11 orbits (from top to bottom)

For quasi-WGMs, the local dispersion can be engineered by RAT, i.e., the tunneling between two regular orbits via island resonance structures in phase space^17,45^, while the global dispersion maintains similar to that of WGMs in a circular cavity. According to Fig. 3a, the integrated dispersion profiles ${D}_{\mathrm{int}}$ of quasi-WGMs clearly exhibit avoided crossing, allowing for fine-tuning of specific resonance frequencies. The avoided mode crossing with splitting 2$g$ can be engineered through RAT, where WGM_m,1_ and WGM_m−10,2_ couple through a period-10 orbit, forming a pair of island (i) and scar (ii) modes, instead of mode families in Fig. 2. Based on the RAT theory described by the pendulum Hamiltonian, the coupling strength $g\propto {A}^{2}$, so that the frequency splitting can be precisely controlled in a wide range by varying the enclosed area $A$ of the stable periodic orbit (Fig. 3b), even exceeding 480 GHz. Note that this theoretical model is applicable not only to near-integrable systems, but also to systems with mixed phase space^46^, which is examined in Fig. S7 of Supplementary Information. Thus, local dispersion can be selectively in-situ engineered by adjusting the enclosed area of the relevant stable periodic orbits (see details in section III of Supplementary Information). For instance, selectively increasing the areas of period-9, -10, or -11 orbits enhances the corresponding frequency splittings on the ${D}_{\mathrm{int}}$ curves (Fig. 3c), while leaving the global dispersion almost unchanged elsewhere. Such in-situ engineering is crucial for applications across multiple wavelength bands, such as multicolor solitons^48,49^.

### Applications for nonlinear optics

**Fig. 4:**
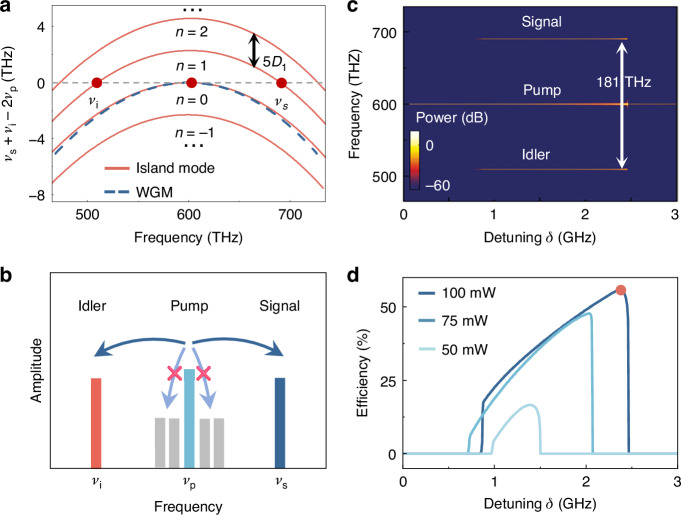
Efficient OPO achieved by the multi-branch global dispersion. **a** Frequency mismatch for the island mode family (red curves) and conventional WGMs (blue dashed curve). The red dots correspond to pump, signal, and idler modes satisfying frequency matching. **b** Illustration of the efficient OPO, showing suppression of parasitic frequency conversion near the pump mode. **c** OPO spectrum of the period-5 island mode family versus the detuning $\delta$. **d** Total conversion efficiency versus detuning under different pump powers

So far, we have presented both global and local dispersion engineering in an ARC, characterized by multi-branch dispersion relations and in-situ controllability, respectively. As a proof of principle for potential applications by global dispersion, efficient third-order OPOs are exhibited based on island modes, targeting the highly desirable blue-violet light^7,50^. Unlike conventional OPOs in microcavities, the tolerance of the momentum matching condition is released via multi-branch dispersion of the island modes, where the mismatch is mitigated by the momentum span among signal, idler, and pump modes. For period-$M$ island modes, the matching condition is ${\mu }_{{\rm{i}}}+{\mu }_{{\rm{s}}}=2{\mu }_{{\rm{p}}}+{nM}\left(N{\mathbb{\in }}{\mathbb{Z}}\right)$, with ${\mu }_{{\rm{i}}}$, ${\mu }_{{\rm{s}}}$, ${\mu }_{{\rm{p}}}$ denoting mode indices of idler, signal, and pump modes, respectively. This relaxed condition enables more flexible frequency matching in the OPO, given by ${\nu }_{{\rm{i}}}+{\nu }_{{\rm{s}}}-2{\nu }_{{\rm{p}}}=\frac{\left({\mu }_{{\rm{s}}}^{2}+{\mu }_{{\rm{i}}}^{2}\right){D}_{2}}{4\pi }+\,\frac{{nM}{D}_{1}}{2\pi }=0$, where the weak high-order dispersion is negligible. Figure 4a shows the frequency mismatch ${\nu }_{{\rm{i}}}+{\nu }_{{\rm{s}}}-2{\nu }_{{\rm{p}}}$ for both period-5 island modes (red solid curves) and WGMs (blue dashed curve). Under globally normal dispersion, OPO cannot emerge in conventional WGMs^23^, yet they are feasible in island modes due to the multi-branch dispersion. The angular momentum span further broadens the frequency separation between signal and idler modes, while the strong normal dispersion suppresses OPO near the pump mode (Fig. 4b). Consequently, with increasing pump power, the conversion efficiency is further improved by overcoming competition from parasitic conversion.

The generation of efficient OPOs is examined based on the experimentally feasible parameters, with thickness 250 nm, deformation parameter $\varepsilon$ = 1/3, an intrinsic (coupling) Q factor of $5\times {10}^{6}$ ($7.14\times {10}^{5}$)^51–54^. The impact of a broad range of quality factors across different modes is further analyzed in Fig. S15 of Supplementary Information. Using the modified Lugiato-Lefever equation incorporating the multi-branch dispersion, the intracavity field spectrum is visualized, with the pump frequency scanned towards red detuning at a pump power of 100 mW (Fig. 4c) (see the calculation details, the stability analysis, and the threshold in section IV of Supplementary Information). Once the OPO threshold is surpassed, the signal light at 691 THz and idler light at 510 THz are observed, yielding a frequency separation exceeding 180 THz. The incracavity powers in the pump, signal, and idler modes grow until reaching a high total efficiency of (*P*_s_ + *P*_i_)/*P*_p_= 56.1% (Fig. 4d), without any conversion into other modes^9,25,55^. In addition to the higher conversion efficiency, the asymmetric microcavity may also help to extract the signal light, achieving a remarkable 600-fold enhancement in the coupling rate between the waveguide and cavity (see details in Fig. S14 of Supplementary Information). This is particularly significant in short-wavelength regimes, where efficient coupling is typically challenging due to the shorter evanescent field.

**Fig. 5:**
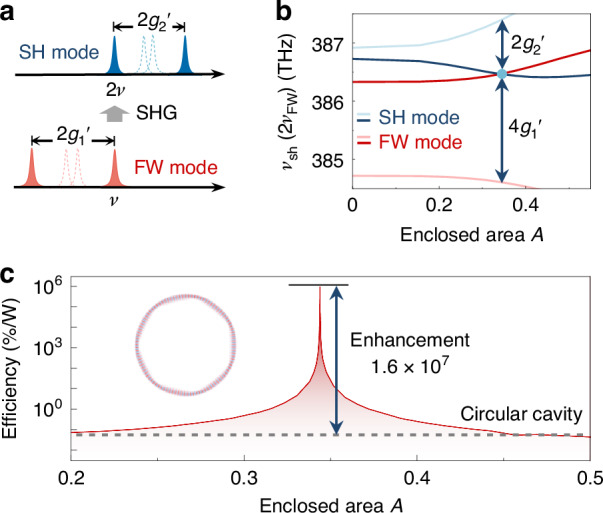
**SHG assisted by the local dispersion engineering**. **a** Schematic spectrum of fundamental and second-harmonic modes for the circular cavity (dashed) and asymmetric cavity (solid). **b** Frequency of SH modes ${\nu }_{{\rm{SH}}}$ and FW modes ${\nu }_{{\rm{FW}}}$ versus the enclosed area $A$ of the period-7 orbit. The blue dot indicates the emergence of double-resonance. **c** Relative conversion efficiency versus the enclosed area $A$, where the grey dashed line represents the efficiency in the circular cavity. Inset: Field distribution of the fundamental mode

As an application of local dispersion engineering, efficient SHG with doubly-resonant enhancement is presented, which is controlled by deformation as a new degree of freedom. As shown in Fig. 5a, the resonance mismatch ${\nu }_{{\rm{SH}}}-2{\nu }_{{\rm{FW}}}$ is finely tuned by RAT-induced modal splitting of both the fundamental-wave (FW) mode 2${g}_{1}^{{\prime} }$, and the second-harmonic (SH) mode 2${g}_{2}^{{\prime} }$. Here, the RAT-induced frequency splitting for FW and SH modes is given by 2${g}_{1(2)}^{{\prime} }=\sqrt{4{g}_{1(2)}^{2}+{\Delta }_{1(2)}^{2}}$, where ${\Delta }_{1(2)}$ denotes the frequency difference between two WGMs from different mode families without deformation, and ${g}_{1(2)}$ is the coupling strength for FW(SH) modes. The momentum matching is fulfilled for the dark blue and red modes in this figure. Using a LiNbO_3_ asymmetric microcavity (see Materials and methods), as the enclosed area $A$ of the stable periodic orbit increases induced by deformation, the resonance mismatching gradually decreases and vanishes at $A\sim 0.34$ (dark-blue and red curves in Fig. 5b). Consequently, a maximum efficiency of $8.5\times {10}^{5}$%/W is achieved when the double resonance condition is satisfied, showing the enhancement of $1.6\times {10}^{7}$ compared to the circular cavity (Fig. 5c).

## Discussion

In summary, we have proposed dispersion engineering in microcavities by symmetry breaking, revealing the multi-branch global dispersion of island modes and in-situ control of local dispersion by RAT. As examples for applications, the efficient OPO is presented with a high efficiency > 55% at blue-violet wave-band, and the efficient SHG is enabled with the double-resonance enhancement. Moreover, this strategy holds remarkable robustness against fabrication imperfections, promoting practical applicability. Looking forward, future work could also explore the extension to different material platforms, potentially unlocking new regimes of light-matter interaction across the visible to ultraviolet spectrum^56–58^.

## Materials and methods

### Calculating global dispersion based on the pendulum Hamiltonian

Considering a series of cavity modes in the same mode family ${\omega }_{{\rm{N}}}$, with $N$ being the mode number relative to the mode ${\omega }_{0}$, the mode frequency is described as

2 $${\omega }_{{\rm{N}}}={\omega }_{0}+{\sum }_{N}\frac{{D}_{{\rm{N}}}{\left(\omega -{\omega }_{0}\right)}^{N}}{N!}$$

where ${D}_{{\rm{N}}}$ is the N-th order mode dispersion coefficient. The ${D}_{{\rm{j}}}$ (*j* = 1,2) can be expressed with *β*_*j*_ as

3 $${D}_{1}=\frac{2\pi }{L{\beta }_{1}},{D}_{2}=-\frac{c}{{n}_{0}}{\left(\frac{2\pi }{L{\beta }_{1}}\right)}^{2}{\beta }_{2}$$

where ${\beta }_{{\rm{j}}}=\frac{{\partial }^{j}k}{\partial {\omega }^{j}}$, *L* is the circumference of light orbit, ${n}_{0}$ is the refractive index, and $c$ is the velocity of light. Therefore, both the dimensions of ${D}_{1}$ and ${D}_{2}$ are 1/s.

The dispersion of island mode families is calculated by perturbation theory^45^, which can speed up the calculation for a large number of resonance modes in an asymmetric microcavity. To this end, the pendulum Hamiltonian $H\left(p,\phi \right)$ is introduced to describe the dynamics in the vicinity of period- $M$ orbits in the phase space, which is expressed as:

4 $$H\left(p,\phi \right)=-\frac{{\left(p-{p}_{{\rm{M}}}\right)}^{2}}{\sqrt{1-{p}_{{\rm{M}}}^{2}}}+2V\left(\varepsilon \right)\cos \left(M\phi +{\phi }_{0}\right)$$

Here ($p=\sin \chi$) are the coordinates in the phase space, ${\phi }_{0}$ is the global phase, and ${p}_{{\rm{M}}}=\cos (\frac{{\rm{\pi }}}{M})$ is the position of stable periodic orbits in the phase space. The coupling strength $V\left(\varepsilon \right)$ reads^45,59^

5 $${\mathcal{V}}\,\left(\varepsilon \right)=\frac{A{\left(\varepsilon \right)}^{2}}{256\sqrt{1-{p}_{{\rm{M}}}^{2}}}$$

where $A\left(\varepsilon \right)$ is the enclosed area of stable periodic orbit. This area $A\left(\varepsilon \right)$ is numerically calculated by fitting the separatrix of the unstable periodic points after canonical transformation^46^. Then, the resonances within an island mode family can be derived from the eigenstate analysis of the following coefficient matrix on the basis of WGMs^45^:

6 $$H=\left[\begin{array}{cccc}\cdots & {\gamma }_{{\rm{M}}}\left({\kappa }_{{\rm{m}}-{\rm{M}},{\rm{q}}+1},{\kappa }_{{\rm{m}},{\rm{q}}}\right){\mathcal{V}}\left(\varepsilon \right) & 0 & 0\\ {\gamma }_{{\rm{M}}}\left({\kappa }_{{\rm{m}}-{\rm{M}},{\rm{q}}+1},{\kappa }_{{\rm{m}},{\rm{q}}}\right){\mathcal{V}}\left(\varepsilon \right) & {\kappa }_{{\rm{m}},{\rm{q}}} & {\gamma }_{{\rm{M}}}\left({\kappa }_{{\rm{m}},{\rm{q}}},{\kappa }_{{\rm{m}}+{\rm{M}},{\rm{q}}-1}\right){\mathcal{V}}\left(\varepsilon \right) & 0\\ 0 & {\gamma }_{{\rm{M}}}\left({\kappa }_{{\rm{m}},{\rm{q}}},{\kappa }_{{\rm{m}}+{\rm{M}},{\rm{q}}-1}\right){\mathcal{V}}\left(\varepsilon \right) & {\kappa }_{{\rm{m}}+{\rm{M}},{\rm{q}}-1} & \cdots \\ 0 & 0 & \cdots & \cdots \end{array}\right]$$

where ${\kappa }_{{\rm{m}},{\rm{q}}}={n}_{{\rm{eff}}}{k}_{{\rm{m}},{\rm{q}}}R,{\gamma }_{{\rm{M}}}({\kappa }_{{\rm{m}},{\rm{q}}},{\kappa }_{{\rm{m}}+{\rm{M}},{\rm{q}}-1})=\mathrm{Re}({n}_{{\rm{eff}}}\frac{{k}_{{\rm{m}},{\rm{q}}}+{k}_{{\rm{m}}+{\rm{M}},{\rm{q}}-1}}{2}R){(2\sqrt{1-{p}_{{\rm{M}}}^{2}})}^{-1}$^45^$,$ with ${n}_{{\rm{eff}}}$ is the effective refractive index, ${k}_{{\rm{m}},{\rm{q}}}={\omega }_{{\rm{m}},{\rm{q}}}/c$, ${\omega }_{{\rm{m}},{\rm{q}}}$ is the frequency of WGM_m,q_ in a circular cavity with the same size ($m,q$ are the azimuthal and radial mode numbers).

The frequency of the island mode family corresponds to the highest eigenvalue of the Hamiltonian. The compositions of the island mode can be extracted according to the eigenstate of this coefficient matrix, with each component representing the amplitude of angular momentum ${a}_{{\rm{N}}}$, thereby accounting for the multi-branch dispersion landscape in Fig. 2d.

### Calculation of SHG based on the RAT

In the investigation of SHG, we use a z-cut lithium niobate micro-disk cavity with radius 13$\,\mathrm{\mu m}$ and thickness 1.3 $\mathrm{\mu m}$. The pump is around 1550 nm, and the intrinsic and coupling quality factors for both fundamental wave (FW) and second-harmonic (SH) modes are$\,5\times {10}^{6}$ with transverse magnetic polarization. The cavity shape follows that $R(\phi )={R}_{0}[1+\varepsilon \cos (7\phi )]$, and thus the local dispersion is in-situ engineered by utilizing a period-7 orbit through RAT. For FW modes, the ${\rm{WG}}{{\rm{M}}}_{{{\rm{m}}}_{{\rm{FW}}},1}$ and ${\rm{WG}}{{\rm{M}}}_{{{\rm{m}}}_{{\rm{FW}}}-7,2}$ couple through the period-7 orbit, where ${m}_{{\rm{FW}}}$ is angular momentum of the FW mode, exhibiting the enlarged mode splitting $2{g}_{1}^{,}=\sqrt{4{g}_{1}^{2}+{\Delta }_{1}^{2}}$ with the increased deformation. Here, ${\Delta }_{1}$ denotes the frequency difference between ${\rm{WG}}{{\rm{M}}}_{{{\rm{m}}}_{{\rm{FW}}},1}$, and ${\rm{WG}}{{\rm{M}}}_{{{\rm{m}}}_{{\rm{FW}}}-7,2}$ without deformation, and ${g}_{1}$ is the coupling strength between the two mode families. For SH modes, the ${\rm{WG}}{{\rm{M}}}_{{{\rm{m}}}_{{\rm{SH}}}+7,2}$ and ${\rm{WG}}{{\rm{M}}}_{{{\rm{m}}}_{{\rm{SH}}},3}$ also couple through the period-7 orbit, presenting similar behavior as FW modes. With the increase of $\varepsilon$ from 0 to 0.005, the enclosed area of the period-7 orbit enlarges from 0 to 0.52 monotonically, and the double-resonance condition is found at $\varepsilon \sim 0.0025$, with the enclosed area $A\sim 0.34$.

## Supplementary information

Supplementary Information for “Dispersion engineering by rotational symmetry breaking in an optical microcavity.”

## Data Availability

The data that support the plots within this paper and other findings of this study are available from the corresponding author upon reasonable request.
