# Supplementary material for: Dispersion engineering by rotational symmetry breaking in an optical microcavity
Source: Light Sci Appl. 2026 Jan 22;15:81. doi: 10.1038/s41377-025-02169-2 (PMC12823584; doi:10.1038/s41377-025-02169-2)
Supplement: Supplementary file 1 — Supplementary Information for “Dispersion engineering by rotational symmetry breaking in an optical microcavity.” [file 41377_2025_2169_MOESM1_ESM.pdf]

Supplementary Information for "Dispersion engineering by rotational symmetry breaking in an optical microcavity"

Jian-Zheng Ren,[1] Li-Jie Li,[1] Rui-Qi Zhang,[1] Zhi-Yan Wang,[1] Qi-Tao Cao,[1] and Yun-Feng Xiao[1,2]

[1] *State Key Lab for Mesoscopic Physics, Frontiers Science Center for Nano-optoelectronics, New Cornerstone Science Lab, School of Physics, Peking University, Beijing 100871, China*

[2] *Collaborative Innovation Center of Extreme Optics, Shanxi University, Taiyuan 030006, China*

This Supplementary text is organized as follows. In section I, boundary of cavities and phase space is illustrated. In section II, dependence of global dispersion on deformation parameters is detailed. In section III, local dispersion engineering for quasi-whispering gallery modes (quasi-WGMs) is analyzed. In section IV, calculation and analysis of optical parametric oscillation (OPO) is elucidated.

I. BOUNDARY OF CAVITIES AND PHASE SPACE

The boundary shape of the asymmetric microcavity used in the main text, a typical and widely studied configuration known as a face cavity [1, 2], is described as:

$$R(\phi) = R_0 + \varepsilon f(\phi) = \begin{cases} R_0 + \varepsilon R_0 \sum_{i=2,3} a_i \cos^i \phi, \cos\phi \geqslant 0 \\ R_0 + \varepsilon R_0 \sum_{i=2,3} b_i \cos^i \phi, \cos\phi < 0 \end{cases} \tag{1}$$

where $R_0 = 50\ \mu m$ is the radius of the cavity, ε is the deformation parameter, and the coefficients are set as $a_2 = -0.1329$, $a_3 = 0.0948$, $b_2 = -0.0642$, $b_3 = -0.0224$.

In classical ray dynamics, the trajectories of light rays in a microcavity are projected on the Poincaré surface of section (PSOS) [2], spanned by the azimuthal angle ϕ and the sine of the incident angle χ, as shown in Fig. S1(a), with the corresponding coordinates in real space shown in Fig. S1(b). Here the deformation parameter is set to ε=1/3. Specifically, the quasi-WGMs in the main text correspond to nearly continuous orbits, *i.e.*, Kolmogorov-Arnol'd-Moser (KAM) curves [red shaded region in Fig. S1(a)], and the island modes are supported by stable periodic orbits [blue shaded region in Fig. S1(a)]. As for the classical rays, different KAM curves are disjoint, while can be coupled together through the resonance-assisted tunneling in wave regimes.

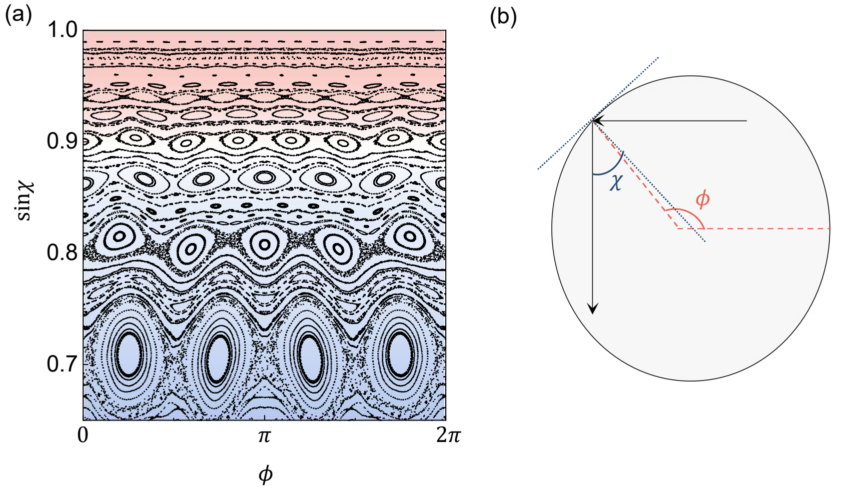

FIG. S1. (a) Phase space of the asymmetric cavity, where the red (blue) shaded region refers to the area of quasi-WGM (island) mode families. (b) Correspondence of phase space coordinates (ϕ, χ) in real space, with blue dashed lines representing the tangent and normal lines of the cavity boundary.

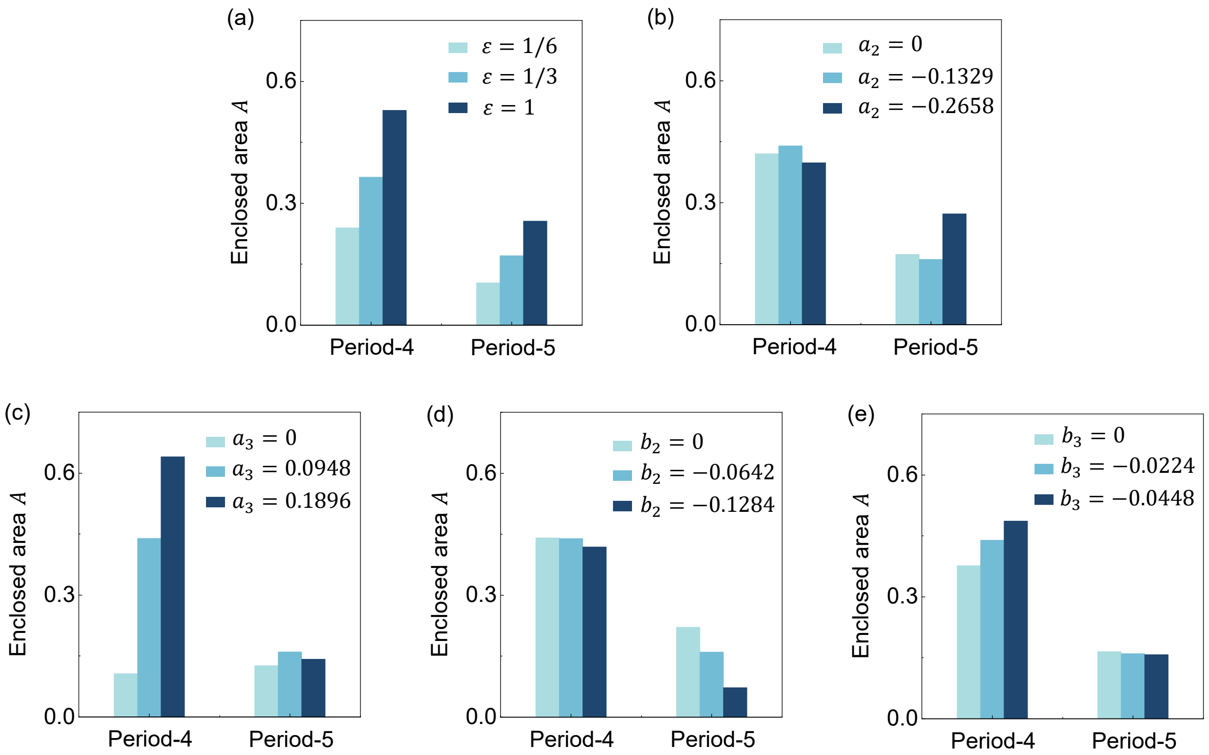

FIG. S2. Enclosed area A for period-4 and 5 orbits for (a) $\varepsilon = 1/6, 1/3, 1$. (b) $a_2 = 0, -0.1329, -0.2658$. (c) $a_3 = 0$, 0.0948, 0.1896. (d) $b_2 = 0, -0.0642, -0.1284$. (e) $b_3 = 0, -0.0224, -0.0448$.

II. DEPENDENCE OF GLOBAL DISPERSION ON DEFORMATION PARAMETERS

In physics, different shapes influence the dispersion by the enclosed area A of stable periodic orbits. As for the global dispersion, the corresponding enclosed area expands the angular momentum span of island mode families. For the face cavity used in this work, the geometry parameters include two categories, i.e., the overall deformation parameter ε and component coefficients a_2, a_3, b_2, b_3. As the deformation parameter ε increases, as shown in Fig. S2(a), the enclosed areas of both period-4 and 5 periodic orbits increase, indicating the increasement of angular momentum span for corresponding island modes. As the component coefficient a_2 decreases, as shown in Fig. S2(b), the variation in enclosed areas of periodic orbits exhibits greater complexity compared to the changes induced by the deformation parameter ε. Basically, the enclosed areas of period-5 orbits first remain nearly constant and then grow rapidly, while the enclosed areas of period-4 orbits remain nearly unchanged. For the increase in component coefficient a_3, as displayed in Fig. S2(c), the enclosed areas of period-5 orbits remain approximately constant, while

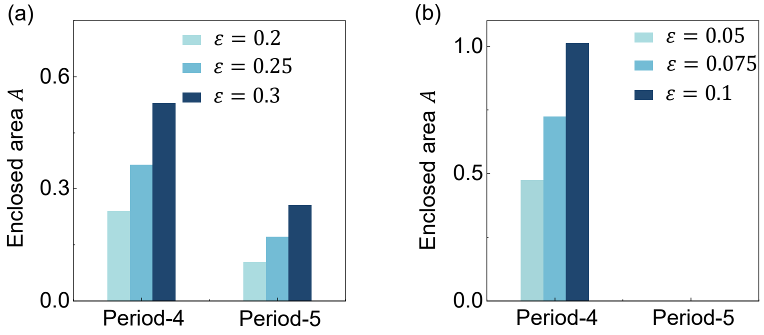

FIG. S3. Enclosed area A for period-4 and 5 orbits for (a) limaçon cavities (ε=0.2,0.25,0.3) and (b) quadrupole cavities (ε=0.05,0.075,0.1).

those of period-4 orbits continuously increase. With the decrease in component coefficient b_2, as shown in Fig. S2(d), the enclosed areas of period-5 continuously decrease, while those of period-4 orbits remains largely unchanged. With the decrease in component coefficient b_3, in Fig. S2(e), unlike the above cases, the enclosed areas of period-4 orbits increase slightly, while period-5 orbits are largely unaffected.

Moreover, our proposed strategy is not only available for a specific cavity shape, but also applicable to other cavity shapes. We further investigate other asymmetric cavities widely studied with different boundary shapes, including limaçon cavity (Fig. S3(a)) and quadrupole cavity (Fig. S3(b))[2], of which the boundary equations read,

$$\text{Limaçon cavity: } R(\phi) = R_0(1 + \varepsilon \cos\phi) \tag{2}$$

$$\text{Quadrupole cavity: } R(\phi) = R_0(1 + \varepsilon \cos 2\phi) \tag{3}$$

For these two asymmetric cavity shapes with different deformation parameter ε, the global dispersion is also determined by the enclosed areas of the short-period orbits. For example, the enclosed areas A of period-4 and 5 orbits in limaçon cavities are increased with the larger deformation ε, as shown in Fig. S3(a), indicating the enhancement of the angular momentum span within the multi-branch dispersion. In the quadrupole cavity, the period-5 orbits are absent due to the geometric symmetry constraint, while the period-4 orbits present the similar behavior for global dispersion engineering as that in limaçon cavities, as shown in Fig. S3(b).

Besides analyzing indirectly mode dispersion through comparing the enclosed area of stable periodic orbits, we then take examples to exhibit the influence of deformation on the mode dispersion directly. In the following, we calculate the integrated dispersion profiles versus geometry parameters ε and a_2 as examples. For the global dispersion of island modes, the integrated dispersion profiles D_{int} are shown in Fig. S4(a) for different deformation parameter ε ($\varepsilon = 1/6, 1/3, 1$). It is found that the fluctuation of D_{int} becomes weaker with the increase of deformation parameter ε due to the stronger coupling between different WGMs with the larger enclosed area of period-5 orbits. Besides, the D_{int} curve exhibits the deviation from a parabola centered at $\mu = 0$, arising from the high-order dispersion over the broad bandwidth ~200 THz for the global dispersion. The angular momentum components for different deformation parameter ε are shown in Fig. S4(b), corresponding to the modes marked by red points in Fig. S4(a). Notably, the span of the angular momentum increases with the deformation parameter ε, agreeing with the increasement of enclosed area of period-5 orbits. For different component coefficients a_2 (a_2=0, -0.1329, -0.2658), as shown in Figs. S4(c) and (d), the dependence of angular momentum span and the fluctuation of D_{int} on a_2 are consistent with the change of enclosed area of period-5 orbits.

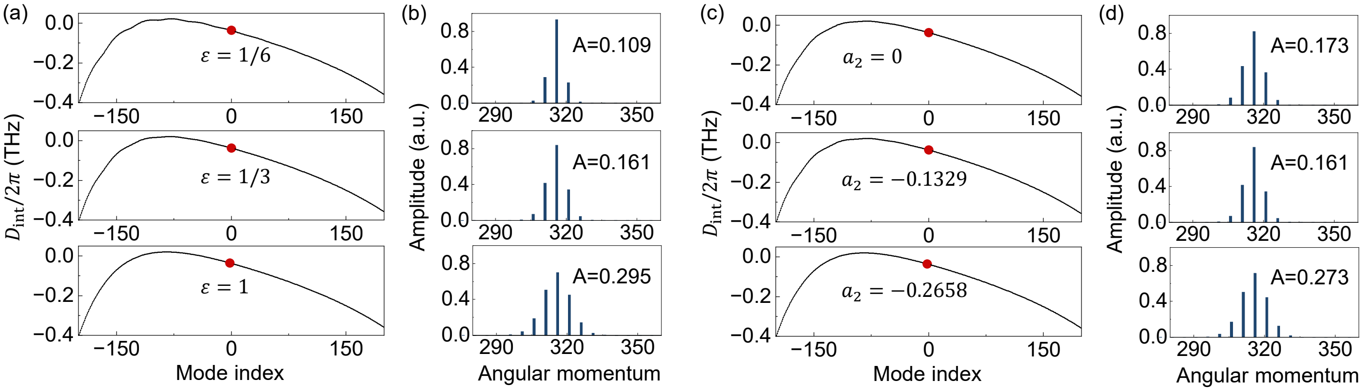

FIG. S4. Integrated dispersion profile D_{int} for period-5 island modes (a) at different deformation parameters (ε=1/6,1/3,1) and (c) at different component coefficients a_2 (a_2=0, a_{20}, $2a_{20}$), a_{20}=-0.1329. (b) (d) Angular momentum components corresponding to the red points in (a) and (c).

III. LOCAL DISPERSION ENGINEERING FOR QUASI-WGMS

A. Local dispersion engineering based on resonance-assisted tunneling

The local dispersion engineering of quasi-WGMs, shown in Fig. S5(a), is induced by resonance-assisted tunneling between two regular orbits via island resonance structures in phase space [also see the whole dispersion curve in Fig.

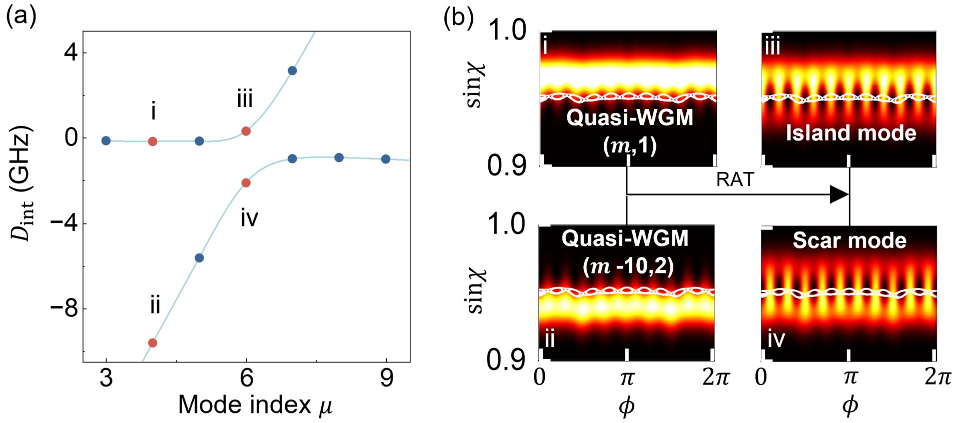

FIG. S5. (a) Integrated dispersion profile $D_{\rm int}$ near the mode crossing with $\varepsilon = 1/3$. (b) Husimi representation for quasi-WGMs (i, ii) and island (scar) mode (iii, iv), referring to the red dots in (a).

3**a**]. The description of this coupling process is illustrated in Fig. S5(b) by using Husimi projection to map the field distribution into the phase space [3]. For quasi-WGMs (m,1) and (m-10,2) away from the coupling region, exemplified as points i and ii, the Husimi projection distributes in different regions of KAM curves separately, divided by period-10 orbits, as shown in the left panels of Fig. S5(b). During the coupling region, the light fields from different KAM curves tunnel into each other and form a pair of island mode (iii) and scar mode (iii), which are localized at the stable and unstable periodic orbits, respectively. Governed by the Fermi resonance condition [4–6], the difference in angular momentum between two quasi-WGMs should equal to the period of the stable periodic orbit.

To in-situ engineer the local dispersion, the deformation function of the asymmetric cavity is designed deliberately. As an example, by introducing the perturbative periodic function $0.001R_0 \cos{(M\phi)}$ $(M = 9, 10, 11)$ selectively, the corresponding Fourier components k of the cavity boundary deformation are enhanced, as shown in Fig. S6(a). In this case, the enclosed area of stable periodic orbits is calculated [Fig. S6(b)], evidently displaying that the area A of the desired period is dominated without affecting others. Therefore, the avoided mode crossing appears at the desired frequency, as illustrated in Fig. 3**c**.

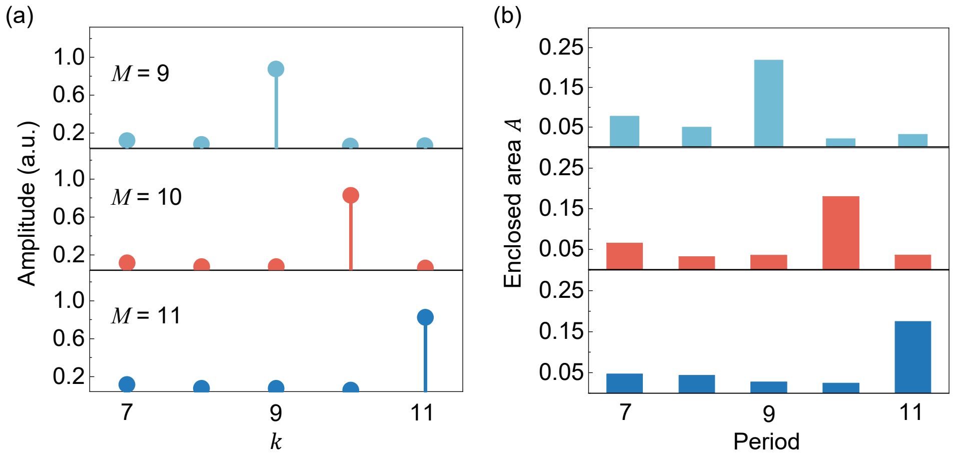

FIG. S6. (a) Spatial Fourier components k of deformation with the selective periodic perturbation. (b) Enclosed area of the stable periodic orbits corresponding to (a).

According to the RAT theory described by the pendulum Hamiltonian, the coupling strength g is proportional to the square of the enclosed area. Quantitively, the relationship between the coupling rate g and the square of enclosed area A^2 of the period-10 stable periodic orbit is examined, through varying deformation parameters ε of the face cavity, as displayed in Figs. S7 (a)(b). The linear dependence between g and A^2 displays good agreement with theoretical predictions by RAT when the deformation parameter $\epsilon < 2.5$. Therefore, the RAT described by the pendulum Hamiltonian is applicable to both near-integrable and mixed phase space. However, under large deformation, the

deviation from the linear prediction is observed at ϵ=2.67. In this case, the emergence of stronger chaos disrupts regular KAM orbits involved in the RAT, as shown in Fig. S7 (c), thus resulting in the failure of RAT predictions.

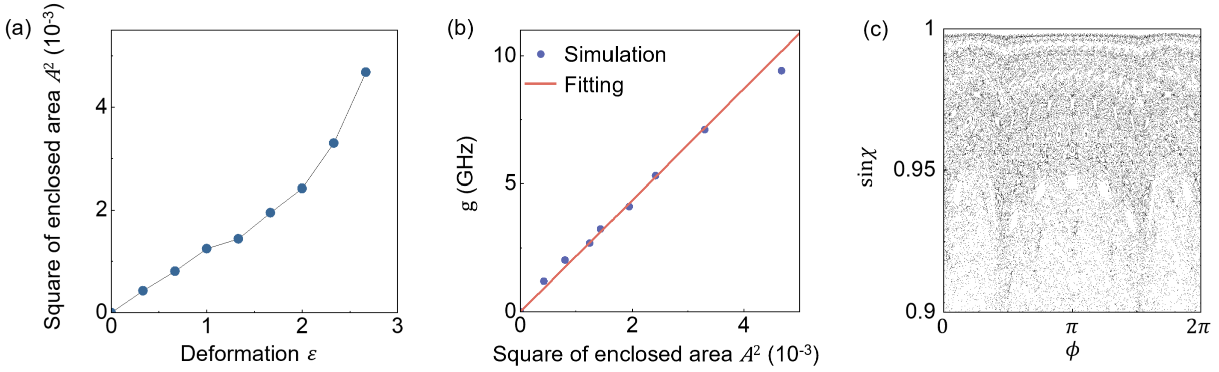

FIG. S7. (a) Square of the enclosed area A^2 of period-10 stable orbit under different deformation ε. (b) Variation of the coupling strength g with the square of the enclosed area A^2 of period-10 stable orbit, with the line and dots represent simulation and fitting data. (c) Phase space of asymmetric cavity with deformation parameter ε=2.67.

B. Dependence of local dispersion on deformation parameters

As for the local dispersion, the enclosed area impacts the strength of resonance-assisted tunneling of quasi-WGMs, thereby changing the coupling strength for the corresponding frequency. We calculate the enclosed area of stable periodic orbits under the same parameters as island modes. The calculation includes long (periods 8, 9, 10 and 11 as four examples) periodic orbits, which determine the local dispersion of quasi-WGMs. As the deformation parameter ε increases, as shown in Fig. S8(a), the enclosed areas of all long periodic orbits increase, indicating the enhancement of coupling strength for quasi-WGMs. As the component coefficient a_2 decreases [Fig. S8(b)], the enclosed areas of odd-period orbits first remain nearly constant and then grow rapidly, and the enclosed areas of even-period orbits remain nearly unchanged. For the increase in the component coefficient a_3 in Fig. S8(c), the enclosed areas of odd-period orbits remain approximately constant, and those of even-period orbits continuously increase. With the decrease in the component coefficient b_2, the enclosed areas of odd-period orbits continuously decrease, and those of

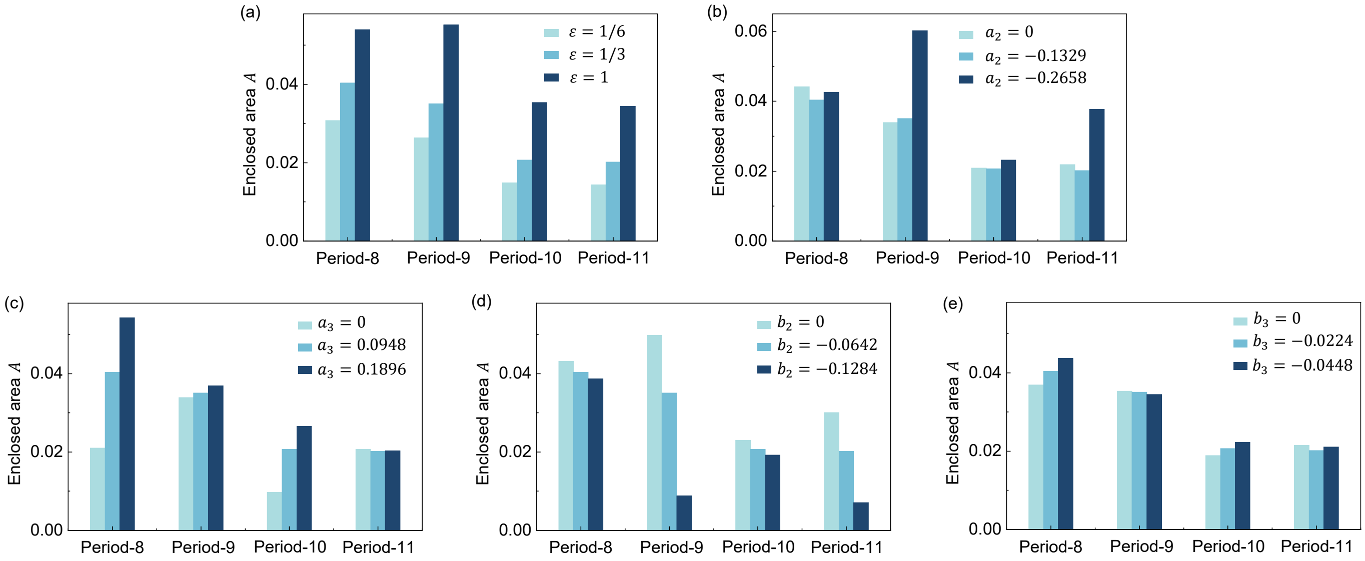

FIG. S8. Enclosed area A for period-8, 9, 10 and 11 orbits for (a) $\varepsilon = 1/6, 1/3, 1$. (b) $a_2 = 0, -0.1329, -0.2658$ (c) $a_3 = 0, 0.0948, 0.1896$. (d) $b_2 = 0, -0.0642, -0.1284$. (e) $b_3 = 0, -0.0224, -0.0448$.

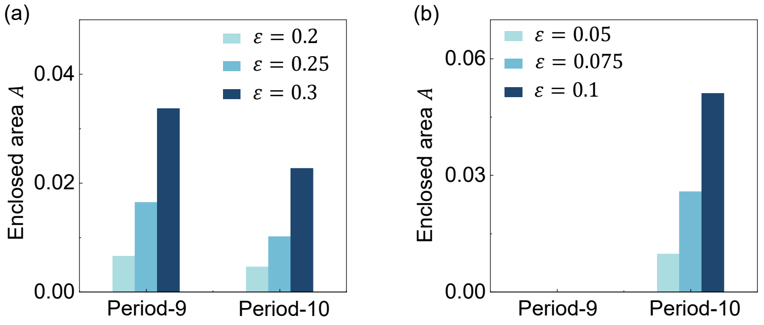

FIG. S9. Enclosed area A for period-9 and 10 orbits for (a) limaçon cavities (ε=0.2, 0.25, 0.3). (b) quadrupole cavities (ε = 0.05, 0.075, 0.1).

even-period orbits remain largely unchanged, as shown in Fig. S8(d). With the decrease in the component coefficient b_3, the enclosed areas of even-period orbits increase slightly, and odd-period orbits are largely unaffected, as displayed in S8(e).

Moreover, the local dispersion strategy is also applicable to other asymmetric cavities widely studied with different boundary shapes, including limaçon cavity and quadrupole cavity, as shown in Fig. S9. For these two asymmetric cavity shapes, local dispersion is also determined by the enclosed areas of long period orbits. For example, the enclosed areas A of each periodic orbits in limaçon cavities are increased with the larger deformation ε, indicating the coupling strength g increasing with deformation. In the quadrupole cavity, the period-9 orbits are absent, and the period-10 orbits present the similar behavior for local dispersion engineering as limaçon cavities.

We then take examples to examine the influence of deformation on local dispersion engineering directly, versus geometry parameters ε and a_2 as examples. For quasi-WGMs, the integrated dispersion profiles D_{int} are shown in Fig. S10(a) for different deformation parameter ε (ε =1/6,1/3,1), where the positions of the engineered local dispersion

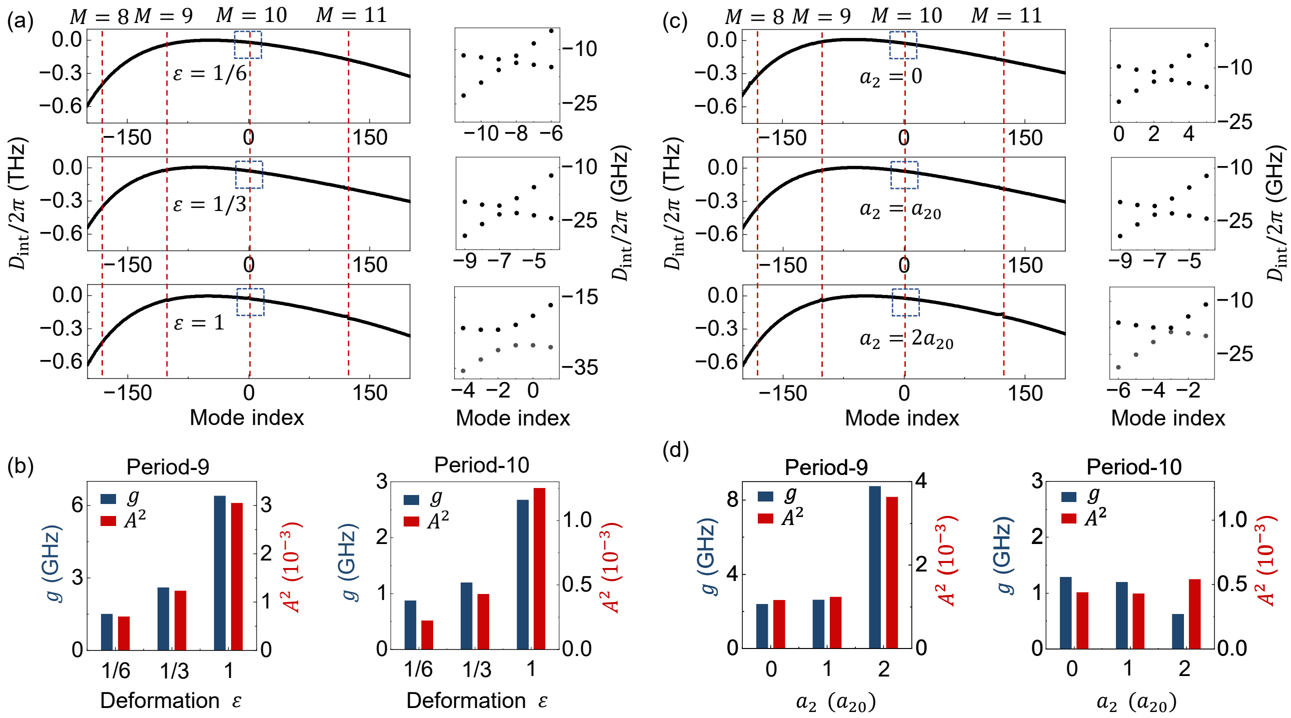

FIG. S10. Integrated dispersion profile D_{int} for quasi-WGM modes (a) at different deformation parameters ε (ε=1/6, 1/3, 1) and (c) at different component coefficients a_2 (a_2=0, a_{20}, $2a_{20}$), a_{20}=-0.1329. Dashed lines correspond to local dispersion controlled by different periodic orbits. Right panel: Zoomed-in view of the engineered local dispersion region of the dashed area. Coupling strength g and the square of enclosed area A^2 of corresponding period-9 and 10 orbits for (b) different deformation parameters ε and (d) different component coefficients a_2.

are marked by the dashed lines. It is found that the overall lineshape of the dispersion curve D_{int} remains nearly unchanged. The details of local dispersion featuring mode crossing controlled by period-10 orbit are shown in the inset of Fig. S10(a), exhibiting that the coupling strength increases with the deformation parameter ε. Quantitatively, the dependence of the frequency splitting in mode crossing on deformation parameter ϵ is shown in Fig. S10(b) for both period-9 and 10 orbits, which agrees with the prediction by the resonance-assisted tunneling theory, i.e., the coupling strength g is proportional to the square of enclosed area of corresponding orbit A^2. For the change of the component coefficient a_2, similar to the results in Figs. S10(a) and (b), the engineering strength of the local dispersion versus a_2 also almost agrees with the prediction by the RAT theory, as shown in Figs. S10(c) and (d).

IV. CALCULATION AND ANALYSIS OF OPO

A. Derivation of calculation of OPO processes

The electric field of the resonance modes inside the cavity can be expressed as [7]:

$$\boldsymbol{E}\left(\boldsymbol{r},t\right)=\sum_{\mu}\frac{1}{2}\varepsilon_{\mu}\left(t\right)e^{i\omega_{\mu}t}\boldsymbol{U}_{\mu}\left(\boldsymbol{r}\right)+\frac{1}{2}\varepsilon_{\mathrm{ext}}\left(t\right)e^{i\Omega_{0}t}\boldsymbol{e}_{0}+c.c. \tag{4}$$

where $\boldsymbol{U}_{\mu}\left(\boldsymbol{r}\right)$ is the electric field of mode with index μ, fulfilling the orthonormalization condition, $\int_V\left[\boldsymbol{U}_{\mu}\left(r\right)\cdot\boldsymbol{U}_{\eta}^{*}\left(r\right)\right]dV=\delta_{\mu,\eta}$, and $\boldsymbol{e}_0$ is the electric field of external pump. ε_{μ} and $\varepsilon_{\mathrm{ext}}$ refer to the amplitude of resonance modes and external pump, respectively. ω_{μ} denotes the resonance frequency, and Ω_0 denotes the frequency of external pump. Taking the Equation (4) into Maxwell Equation:

$$\left[\Delta-\frac{\epsilon\left(\boldsymbol{r},\omega,|\boldsymbol{E}|^{2}\right)}{c^{2}}\frac{\partial^{2}}{\partial t^{2}}\right]\boldsymbol{E}(\boldsymbol{r},t)=0,\quad \epsilon\left(\boldsymbol{r},\omega,|\boldsymbol{E}|^{2}\right)=\begin{cases}n_{\mathrm{tot}}^{2}\left(\omega,|\boldsymbol{E}|^{2}\right),r\leqslant R(\theta)\\ 1,r>R(\theta)\end{cases} \tag{5}$$

ϵ is the permittivity and n_{tot} is the refractive index considering the modulation of electric fields and frequency, thus we can obtain

$$\left[\Delta-\frac{\epsilon\left(\boldsymbol{r},\omega,|E|^{2}\right)}{c^{2}}\frac{\partial^{2}}{\partial t^{2}}\right]\left(\sum_{\mu}\frac{1}{2}\varepsilon_{\mu}\left(t\right)e^{i\omega_{\mu}t}\boldsymbol{U}_{\mu}\left(r\right)+\frac{1}{2}\varepsilon_{\mathrm{ext}}\left(t\right)e^{i\Omega_{0}t}\boldsymbol{e}_{0}+c.c.\right)=0. \tag{6}$$

The permittivity ϵ under the perturbation is expressed as:

$$\epsilon\left(\omega,|\boldsymbol{E}|^{2}\right)=\left[n_{0}\left(\omega\right)+\Delta n_{\mathrm{tot}}\left(\omega,|\boldsymbol{E}|^{2}\right)\right]^{2}\approx n_{0}^{2}\left(\omega\right)+2n_{0}(\Omega_{0})\Delta n_{\mathrm{tot}}\left(\omega,|\boldsymbol{E}|^{2}\right) \tag{7}$$

Here $n_0(\Omega_0)$ is the refractive index of silicon nitride at Ω_0 which is simplified as n_0 below (in this paper $n_0=2.04$). Δn_{tot} includes two parts: one is the intrinsic (n_{int}) and external loss (n_{ext}), and the other is the cross-phase modulation, depending on the amplitude of the electric field, *i.e.*,

$$\Delta n_{\mathrm{tot}}\left(\omega,|\boldsymbol{E}|^{2}\right)=-i\left[n_{\mathrm{int}}\left(\omega\right)+n_{\mathrm{ext}}\left(\omega\right)\right]+n_{2}\frac{n_{0}\varepsilon_{0}c}{2}|\boldsymbol{E}|^{2}=-i\left[n_{\mathrm{int}}\left(\omega\right)+n_{\mathrm{ext}}\left(\omega\right)\right]+n_{2}\frac{n_{0}\varepsilon_{0}c}{2}\sum_{m,n}\varepsilon_{m}\varepsilon_{n}^{*}e^{i(\omega_{m}-\omega_{n})t}\boldsymbol{U}_{m}\boldsymbol{U}_{n}^{*} \tag{8}$$

Here $n_2=2.4\times10^{-19}$ m^2/W [8] is the nonlinear coefficient of silicon nitride. Using the slowly varying amplitude approximation: $|\ddot{\varepsilon}_{\mu}\left(t\right)|\ll|\omega_{\mu}\dot{\varepsilon}_{\mu}\left(t\right)|,|\dot{\varepsilon}_{\mu}\left(t\right)|\ll|\omega_{\mu}\varepsilon_{\mu}\left(t\right)|$, and substituting equation (7) into (6), we have:

$$\sum_{\mu}\omega_{\mu}\dot{\varepsilon}_{\mu}\left(t\right)e^{i\omega_{\mu}t}\boldsymbol{U}_{\mu}\left(r\right)=-\sum_{\mu}i\omega_{\mu}^{2}\frac{n_{0}\Delta n_{\mathrm{tot}}\left(\omega,|E|^{2}\right)}{\epsilon\left(\boldsymbol{r},\Omega_{0},0\right)}\varepsilon_{\mu}\left(t\right)e^{i\omega_{\mu}t}\boldsymbol{U}_{\mu}\left(r\right)-\frac{1}{2}i\Omega_{0}^{2}\varepsilon_{\mathrm{ext}}e^{i\Omega_{0}t}\boldsymbol{e}_{0} \tag{9}$$

The equation (9) is then multiplied by $\boldsymbol{U}_{\eta}\left(r\right)$ and integrated over the whole space using the orthonormalization condition, yielding

$$\omega_{\eta}\dot{\varepsilon}_{\eta}\left(t\right)=-\int_{V}\sum_{\mu}i\omega_{\mu}^{2}\frac{n_{0}\Delta n\left(\omega,|\boldsymbol{E}|^{2}\right)}{\epsilon\left(\boldsymbol{r},\Omega_{0},0\right)}\varepsilon_{\mu}\left(t\right)e^{i\omega_{\mu}t-i\omega_{\eta}t}\left[\boldsymbol{U}_{\mu}\left(r\right)\cdot\boldsymbol{U}_{\eta}^{*}\left(r\right)\right]dV-\int_{\infty}\frac{1}{2}i\Omega_{0}^{2}\varepsilon_{\mathrm{ext}}e^{i(\Omega_{0}-\omega_{\eta})t}\left[\boldsymbol{e}_{0}\cdot\boldsymbol{U}_{\eta}^{*}\left(r\right)\right]dV. \tag{10}$$

Under the normalization $A_\eta = \sqrt{\frac{\varepsilon_0 n_0^2}{2\hbar\omega_\eta}}\varepsilon_\eta$, $F_\eta = -\frac{i\Omega_0^2}{\omega_\eta\kappa_\eta}\sqrt{\frac{\varepsilon_0 n_0^2}{2\hbar\omega_\eta}}\varepsilon_{\text{ext}}\int_\infty \boldsymbol{e}_0\cdot\boldsymbol{U}_\eta^*(r)\,dV$, and $\kappa_\eta = \kappa_{\text{ext},\eta}+\kappa_{\text{int},\eta}$, $g_0 = \frac{n_2 c}{n_0^2}\frac{\hbar\omega_{\eta0}^2}{V_{\eta0}}$, $V_{\eta0} = \left[\int_V \|U_{\eta0}(r)\|^4 dV\right]^{-1}$ where η_0 is the pump mode, $\Lambda_\eta^{\alpha\beta\mu} = \frac{\omega_\mu^2}{\omega_{\eta0}^2}\sqrt{\frac{\omega_\alpha\omega_\beta\omega_\mu}{\omega_\eta^3}}\frac{\int_V\left[\boldsymbol{U}_\mu(r)\cdot\boldsymbol{U}_\eta^*(r)\right]\left[\boldsymbol{U}_\alpha(r)\cdot\boldsymbol{U}_\beta^*(r)\right]dV}{\int_V\|\boldsymbol{U}_{\eta0}(r)\|^4 dV}$, $\omega_{\alpha\beta\mu\eta} = \omega_\alpha-\omega_\beta+\omega_\mu-\omega_\eta$, equation (10) is simplified into:

$$\dot{A}_\eta = -\frac{1}{2}\kappa_\eta A_\eta - ig_0\sum_{\alpha,\beta,\mu}\Lambda_\eta^{\alpha\beta\mu}A_\alpha A_\beta^* A_\mu e^{i\omega_{\alpha\beta\mu\eta}} + \frac{1}{2}\kappa_\eta F_\eta e^{i(\Omega_0-\omega_\eta)t} \tag{11}$$

Defining that $B_\eta = A_\eta e^{i(\omega_\eta-\Omega_0)t} = A_\eta e^{i(\omega_\eta-\omega_0)t-i\sigma t}, \sigma = \Omega_0-\omega_{\eta0}$, the equation (11) becomes:

$$\dot{B}_\eta = -\frac{1}{2}\kappa_\eta B_\eta - ig_0\sum_{\alpha,\beta,\mu}\Lambda_\eta^{\alpha\beta\mu}B_\alpha B_\beta^* B_\mu + i(\omega_\eta-\omega_0-\sigma)B_\eta + \frac{1}{2}\kappa_\eta F_\eta\delta_{\eta,\eta_0}. \tag{12}$$

For the cavity with rotational symmetry, the momentum matching is equivalent to the mode index matching, which means that $\Lambda_\eta^{\alpha\beta\mu}\neq 0$ equals to $\alpha+\mu=\beta+\eta$. While for island mode families in asymmetric cavities, due to the multi-branch dispersion, the momentum matching does not necessarily require the conservation of the mode index. When $\alpha+\mu-\beta-\eta = nM$ (n is the order of phase matching and M is the period of island mode family), $\Lambda_\eta^{\alpha\beta\mu}\neq 0$. Approximately, for an island mode family, when $\alpha+\mu-\beta-\eta=nM$, $\Lambda_\eta^{\alpha\beta\mu}\approx\Lambda_n$, $\Lambda_0\approx 1$. Therefore, the equation (12) is simplified into:

$$\dot{B}_\eta = -\frac{1}{2}\kappa_\eta B_\eta - ig_0\sum_{\alpha,\beta,\mu}\Lambda_n B_\alpha B_\beta^* B_\mu\delta(\alpha+\mu-\beta-\eta-nM) + i(\omega_\eta-\omega_0-\sigma)B_\eta + \frac{1}{2}\kappa_\eta F_\eta\delta_{\eta,\eta_0} \tag{13}$$

Rewriting that $B(\varphi,t) = \sum_\eta B_\eta e^{i(\eta-\eta_0)\varphi}$ and neglecting the variation of κ_η ($\kappa_\eta\approx\kappa$), the equation (13) can be transformed into:

$$\begin{aligned}\frac{\partial B(\varphi,t)}{\partial t} &= \sum_\eta\left[-\frac{1}{2}\kappa B_\eta e^{i(\eta-\eta_0)\varphi} + i(\omega_\eta-\omega_0-\sigma)B_\eta e^{i(\eta-\eta_0)\varphi}\right]\\ &\quad - ig_0\sum_{\alpha,\beta,\mu,n}B_\alpha B_\beta^* B_\mu\Lambda_n\delta(\alpha+\mu-\beta-\eta-nM)e^{i(\eta-\eta_0)\varphi} + \frac{1}{2}\kappa F_0\end{aligned} \tag{14}$$

Here φ is an intermediate variable in Fourier transformation and denotes the Fourier conjugation of the mode number μ. Given that $\omega_\eta-\omega_0\approx(\eta-\eta_0)D_1+\frac{(\eta-\eta_0)^2}{2}D_2$, $\frac{\partial B(\varphi,t)}{\partial\varphi} = \sum_\eta i(\eta-\eta_0)B_\eta e^{in\varphi}$, $\frac{\partial^2 B(\varphi,t)}{\partial\varphi^2} = -\sum_\eta(\eta-\eta_0)^2 B_\eta e^{i(\eta-\eta_0)\varphi}$, the Equation (14) can be expressed as:

$$\frac{\partial B(\varphi,t)}{\partial t} = \left(-i\sigma-\frac{1}{2}\kappa\right)B(\varphi,t) + \left(D_1\frac{\partial}{\partial\varphi} - i\frac{D_2}{2!}\frac{\partial^2}{\partial\varphi^2}\right)B(\varphi,t) - ig_0|B(\varphi,t)|^2 B(\varphi,t)\sum_n\Lambda_n e^{in\varphi} + \frac{1}{2}\kappa F_{\eta_0} \tag{15}$$

where $F_{\eta_0} = \sqrt{\frac{4\kappa_{\text{ex}}P_{\text{in}}}{\hbar\omega_0\kappa^2}}$ .While for $n>2$, $\Lambda_n\ll 1$ which can be neglected, and $\Lambda_n\approx\Lambda_{-n}$, the equation (15) is further simplified as:

$$\begin{aligned}\frac{\partial B(\varphi,t)}{\partial t} &= \left(-i\sigma-\frac{1}{2}\kappa\right)B(\varphi,t) + \left(D_1\frac{\partial}{\partial\varphi} - i\frac{D_2}{2!}\frac{\partial^2}{\partial\varphi^2}\right)B(\varphi,t)\\ &\quad - ig_0|B(\varphi,t)|^2 B(\varphi,t)\left(1+2\Lambda_1\cos(\varphi)+2\Lambda_2\cos(2\varphi)\right) + \frac{1}{2}\kappa F_0\end{aligned} \tag{16}$$

Equation (16) is the modified Lugiato-Lefever equation incorporating the multi-branch dispersion. For the period-5 island mode considering in the main text, $\Lambda_1\approx 0.437, \Lambda_2\approx 0.208$, which is calculated using the finite element methods.

The detailed calculation parameters for the OPO efficiencies and frequency separations are given below. The first-order dispersion coefficients $D_1/2\pi$ for period-5 around 600 THz is 458.7 GHz. The second-order dispersion coefficients $D_2/2\pi$ is -58.7 MHz. The spectrum is calculated by the inverse Fourier transformation $B_\mu(t) = \frac{1}{2\pi}\int_0^{2\pi}B(\varphi,t)e^{-i\varphi(\mu-\mu_0)}d\varphi$, where the intermediate variable φ is eliminated.

Below the threshold, according to equation (13), the pump mode obeys

$$-\frac{1}{2}\kappa B_0 - ig_0\left|B_0\right|^2 B_0 - i\sigma B_0 + f = 0 \tag{17}$$

where $f = \sqrt{\frac{\kappa_{\text{ex}}P_{\text{in}}}{\hbar\omega_0}}$. For a pair of sidebands $-\mu$ and l satisfying momentum and energy matching condition, their value $B_{-\mu,l} = 0$ below the threshold of OPO. $\delta B_{-\mu,l}$ are introduced to perturb the equilibrium to perform a linear stability analysis, and the pump power when the perturbation grows exponentially is defined as threshold:

$$\delta\dot{B}_l = \left(-\frac{1}{2}\kappa - 2ig_0\left|B_0\right|^2 - i\sigma - \frac{i}{2}\left(2\omega_0 - \omega_{-\mu} - \omega_l\right)\right)\delta B_l - ig_0\Lambda_1 {B_0}^2 \delta {B_{-\mu}}^* \tag{18}$$

$$\delta\dot{B}_{-\mu} = \left(-\frac{1}{2}\kappa - 2ig_0\left|B_0\right|^2 - i\sigma - \frac{i}{2}\left(2\omega_0 - \omega_{-\mu} - \omega_l\right)\right)\delta B_{-\mu} - ig_0\Lambda_1 {B_0}^2 \delta {B_l}^* \tag{19}$$

The equation (18) and (19) can be rearranged into

$$\begin{pmatrix} \delta\dot{B}_l \\ \delta\dot{B}^*_{-\mu} \end{pmatrix} = T \begin{pmatrix} \delta B_l \\ \delta {B_{-\mu}}^* \end{pmatrix}, \tag{20}$$

When the real part of the largest eigenvalue of matrix T becomes larger than 0, OPO occurs, corresponding to

$$\kappa^2 + \left(4g_0\left|B_0\right|^2 + 2\sigma + \left(2\omega_0 - \omega_{-\mu} - \omega_l\right)\right)^2 - 4{g_0}^2\Lambda_1^2\left|B_0\right|^4 < 0 \tag{21}$$

Substituting Equation (17) into (21), the minimum of $|B|^2$ is calculated as

$$\left|B_{\text{min}}\right|^2 = \frac{\kappa}{2\Lambda_1 g_0} \tag{22}$$

Substitute equation (22) into (17), the corresponding pump power

$$P = \frac{\hbar\omega_0}{\kappa_{\text{ex}}}\left[{g_0}^2\left(\frac{\kappa}{2\Lambda_1 g_0}\right)^3 + 2\sigma g_0\left(\frac{\kappa}{2\Lambda_1 g_0}\right)^2 + \left(\left(\frac{1}{2}\kappa\right)^2 + \sigma^2\right)\frac{\kappa}{2\Lambda_1 g_0}\right] \tag{23}$$

The threshold power corresponds to the minimum power value under different detuning σ

$$P_{\text{th}} = \frac{\hbar\omega_0(\kappa_0 + \kappa_{\text{ex}})^3}{8g_0\Lambda_1\kappa_{\text{ex}}} \tag{24}$$

The pump threshold of OPO against the coupling rate κ_{ex} is then calculated, shown in Fig. S11, where the lowest threshold power 3.51 mW is found at $\kappa_{\text{ex}} = 1/2\kappa_0$ (red point). When the critical coupling condition $\kappa_{\text{ex}} = \kappa_0$ is satisfied, the threshold power is 4.16 mW (orange point).

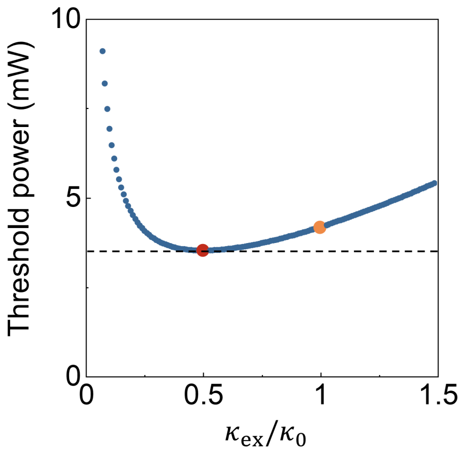

FIG. S11. Variation of threshold power with κ_{ex}. The red and orange dots represent the minimum threshold power and the threshold power at critical coupling, respectively.

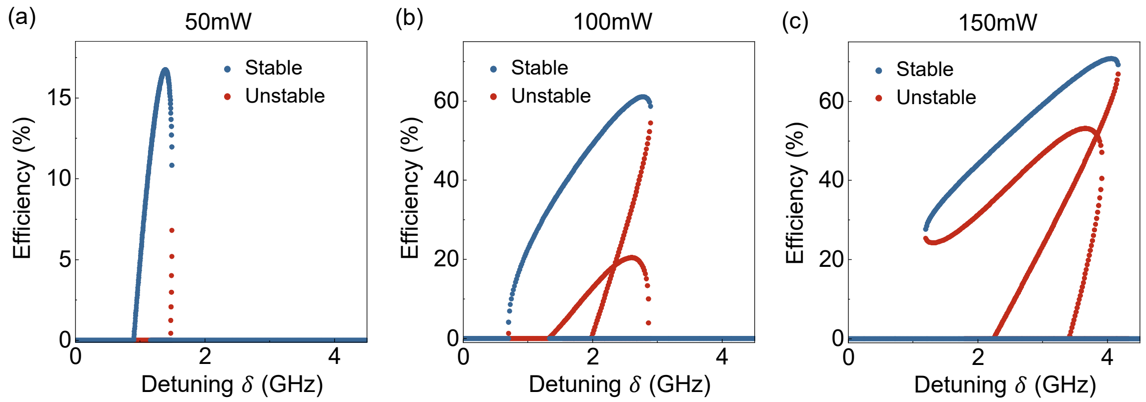

FIG. S12. (a) to (c) Analysis of OPO generation efficiency at stable and unstable solutions (blue and red points) under the three-mode coupling equations, corresponding to pump powers of 50, 100, and 150 mW.

B. Stability analysis of OPO processes

The stability analysis of the OPO processes is conducted based on three-modes coupled equations with the parameters described in the main text:

$$\begin{aligned} \dot{a}_p &= \left(-i\sigma - \frac{1}{2}\kappa - ig_0(2|a_s|^2 + 2|a_t|^2 + |a_p|^2)\right) a_p - 2ig_0\Lambda_1 a_s a_t a_p^* + \frac{1}{2}\kappa F_0 \\ \dot{a}_s &= \left(i(\omega_s - \omega_0 - \sigma) - \frac{1}{2}\kappa - ig_0(|a_s|^2 + 2|a_t|^2 + 2|a_p|^2)\right) a_s - ig_0\Lambda_1|a_p|^2 a_i^* \\ \dot{a}_i &= \left(i(\omega_t - \omega_0 - \sigma) - \frac{1}{2}\kappa - ig_0(2|a_s|^2 + |a_t|^2 + 2|a_p|^2)\right) a_i - ig_0\Lambda_1|a_p|^2 a_s^* \end{aligned} \tag{25}$$

Specifically, the steady-state solutions of the three-mode coupled equations are first calculated numerically, where the pump mode is set at 500 nm. Subsequently, the stability of each steady-state solution is distinguished by analyzing the eigenvalues of the corresponding Jacobian matrix J [7], defined as:

$$\begin{pmatrix} \dot{a}_p \\ \dot{a}_s \\ \dot{a}_i \end{pmatrix} = J \begin{pmatrix} \delta a_p \\ \delta a_s \\ \delta a_i \end{pmatrix} \tag{26}$$

where δa_p, δa_s and δa_i is the perturbation of the steady-state solutions. The instability occurs if J has eigenvalues with positive real parts at the steady-state solution, while the stability holds otherwise. Using this criterion, the efficiencies of the OPO with both instability and stability are calculated during scanning from blue to red detuning under different laser powers.

At the pump power slightly higher the threshold [e.g., 50 mW in Fig. S12(a)], the system first enters the stable OPO state, then transitions to bistable states. Ultimately, the light field only remains in the pump mode. At higher power [100 mW in Fig. S12(b)], the behavior resembles the stability at 50 mW but with additional unstable OPO solutions in the bistable region. Notably, three-mode calculations predict 61% efficiency, exceeding LLE simulation in the revised manuscript due to the neglected competing processes. When further increasing the pump power [150 mW in Fig. S12(c)], the pump mode remains stable during scanning, and stable OPO state cannot be directly accessed through frequency scanning.

C. Comparison to OPOs by conventional dispersion engineering

Our work presents an available solution to the long-standing challenges of OPOs, especially in the blue-violet light spectrum. By leveraging the dispersion engineering by rotational symmetry breaking, we have predicted the record-high efficiency while overcoming three major challenges [9–13]. **(1) Generation of OPOs**: Conventional photonic materials suffer from severe dispersion in the blue-violet band due to proximity to the absorption edge. Our

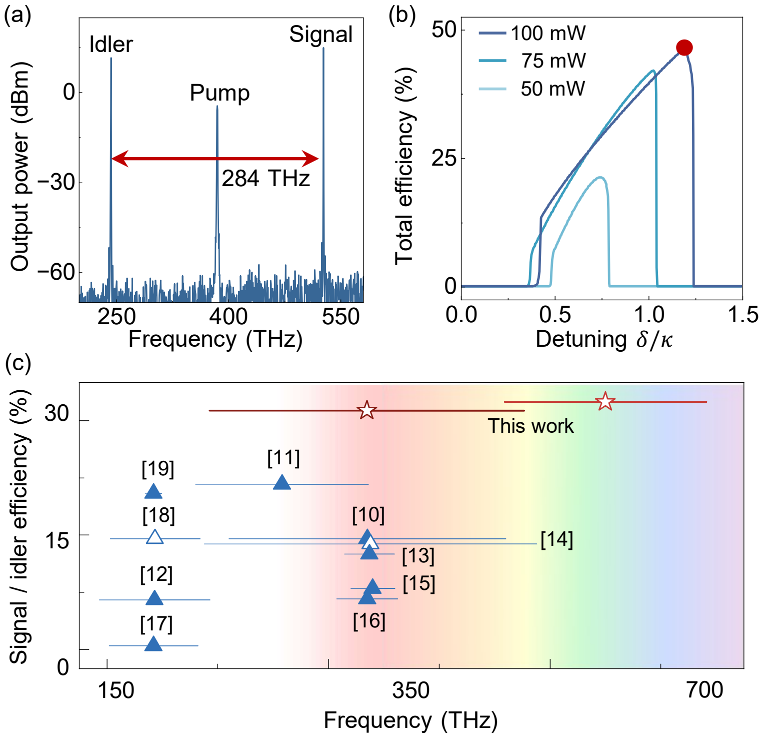

FIG. S13. (a) Output OPO spectra at pump power 100 mW and detuning δ=1.19 GHz. (b) Total conversion efficiency versus detuning under different pump powers. The red dot corresponds to (a). (c) Comparison of signal or idler conversion efficiency and spectral coverage between the OPOs by this work and previously reported results [10–19]. The hollow (solid) symbols mark the theoretical (experimental) results.

approach utilizes the multi-branch dispersion of island mode families to compensate for this frequency mismatch, enabling efficient phase matchinga, as shown in Fig. 4**a** in the main text. **(2) Efficiency of OPOs**: Conventional single-mode-family OPOs suffer from parasitic oscillations near the pump mode under high intracavity power. We circumvent this issue by employing a novel dispersion engineering strategy that maintains strong normal dispersion across pump, signal, and idler modes, ensuring stable and efficient parametric conversion, as shown in Fig. 4**b** in the main text. **(3) Extraction of OPOs**: Short-wavelength light fields typically exhibit low extraction efficiency due to their limited evanescent field overlap. Our design exploits the enhanced evanescent fields of island modes at specific locations [Fig. S14(b)], significantly improving waveguide-cavity coupling rate. Consequently, it is calculated that the coupling Q factor can reach 7×10^5 for the island mode at 691 THz with gap distance 170 nm [Fig. S14(c)], corresponding to the strong over-coupling condition $\kappa_{\rm ex} = 7\kappa_0$.

Besides, considering the current absence of blue-light OPOs for a direct comparison with our work, we would further examine our proposed mechanism in $\sim$ 780 nm bands to facilitate a comparison with existing literatures [10, 13–16]. In this calculation, the thickness of the cavity is 700 nm, and the deformation is expressed as $R(\phi) = R_0[1+1/2f(\phi)]$. The cavity-waveguide coupling Q factor is 1.25×10^6, and other parameters are same as that in Fig. 4 in the main text. Once the threshold is surpassed, the OPO emerges with signal light at 527 THz (569 nm) and idler light at 243 THz (1235 nm), yielding a frequency separation 284 THz (666 nm), as shown in Fig. S13 (a). The first and second-order dispersion coefficients $D_1/2\pi$ and $D_2/2\pi$ are 486.0 Ghz and -28.4 MHz. Moreover, a record-high total conversion efficiency is also found, i.e., $(P_s + P_i)/P_p = 46.1\%$ with pump power 100 mW, as shown in Fig. S13 (b). In view of the previous literatures [Fig. S13 (c)], our theoretical approach could offer the potential to surpass the maximum conversion efficiency reported in previous studies while achieving a remarkably large frequency separation. Moreover, our scheme is particularly advantageous for over-coupled designs in the short-wavelength regime, which is critical for efficient frequency conversion in the visible band.

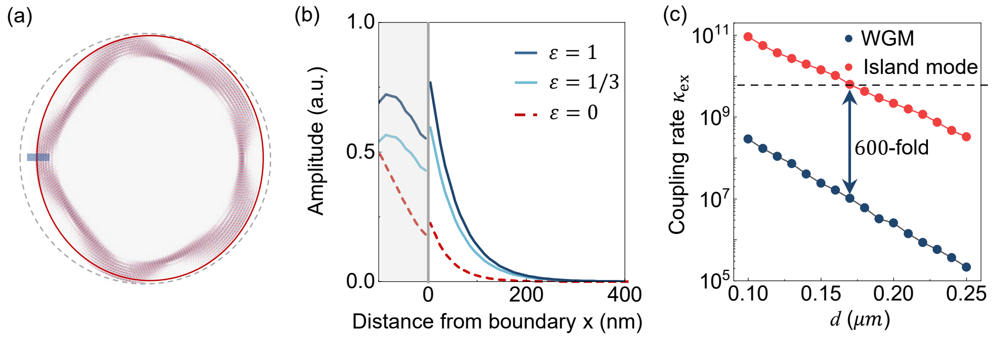

FIG. S14. (a) Typical field distribution of a period-5 island mode in the asymmetric cavity. (b) Evanescent field distribution near the boundary in the mark region of (a), where the shaded region represents the area in the cavity. (c) Coupling rate κ_{ex} against the gap d between the cavity and waveguide. Red and blue points denote the κ_{ex} of the island mode in an asymmetric cavity and WGM in a circular cavity respectively, and the dashed line corresponds to $Q \sim 7 \times 10^5$ for the extraction of blue light.

D. Coupling between cavity and waveguide in asymmetric cavities

For the coupling between cavity and waveguide, asymmetric cavity modes can have stronger evanescent field at particular position due to the anisotropic distribution. Besides the stronger evanescent coupling quasi-WGMs reported before [20], the normalized field distribution ($\int |E|^2 ds = 1$) near the boundary in the island modes (Figs. S14(a)(b)) shows that the evanescent field is significantly enhanced compared to that of the circular cavity mode (TE_0 modes near 691 THz as an example). This implies that the island modes exhibit stronger coupling with the waveguide compared to conventional WGM modes. Furthermore, the enhanced evanescent field under larger deformation indicates increased coupling strength, resulting in a decreased coupling Q-factor. The waveguide coupling is designed by placing the waveguide at the position of maximum evanescent field intensity, in the shaded region of Fig. S14(a), with the width of waveguide 160 nm and the thickness the same as cavity. Based on the calculated field, the coupling rate κ_{ex} between the waveguide and cavity is quantitatively obtained through field overlap, shown as red points in Fig. S14(c). The requirement of coupling $Q \sim 7 \times 10^5$ for the extraction of blue light can be acquired in the island mode [the dashed line in Fig. S14(c)], at an experimentally feasible gap distance of 170 nm. As a contrast, the coupling rate κ_{ex} for the circular cavity at the same wavelength is calculated and found to be 600-fold weaker than that of the island mode. For the pump and idler modes, the larger evanescent field makes over-coupling condition easier to achieve than at shorter wavelengths.

E. Conversion efficiency for a broad range of quality factors

In realistic material systems, intrinsic quality factors (Q) differ across spectral bands due to variations in absorption and scattering. To broaden the applicability of our theory, we evaluate the OPO conversion efficiency with varying intrinsic Q among the signal (Q_{s}), pump (Q_{p}), and idler (Q_{i}) modes. Specifically, Q_{s} is varied from 10^5 to 10^7, Q_{i} from 5×10^5 to 10^7, and Q_{p} is fixed at 5×10^6. The coupling quality factors are set to 1.25×10^5, 2.5×10^6, and 1.25×10^6 for signal, idler, and pump, respectively. The quality factors of the other modes are obtained through interpolation functions, and other parameters are consistent with those in Fig. 4 of the main text under a 100 mW pump power. As shown in Fig. S15(a), our scheme remains robust and operates efficiently under these experimentally feasible parameters. Quantitatively, efficient conversion exceeding 50% can still be achieved with $Q_{\mathrm{s}} \sim 5 \times 10^5$ and $Q_{\mathrm{i}} > 3 \times 10^6$, both of which are typically achievable in practical devices [21], as shown in Fig. S15(b). The coupling quality factor of the signal mode is 1.25×10^5, which can be achieved with a waveguide-cavity gap of 120 nm (which is calculated in Fig. S14(c))—well within the capabilities of current nanofabrication technology.

[1] X. Jiang, L. Shao, S.-X. Zhang, X. Yi, J. Wiersig, L. Wang, Q. Gong, M. Lončar, L. Yang, and Y.-F. Xiao, Chaos-assisted broadband momentum transformation in optical microresonators, Science **358**, 344 (2017).

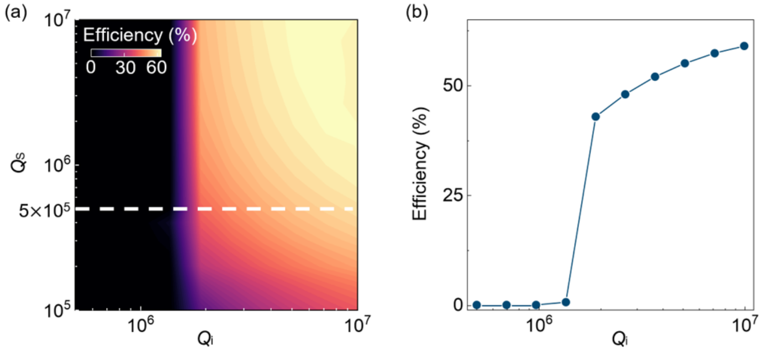

FIG. S15. (a) Conversion efficiency versus the intrinsic quality factors of signal mode Q_s and idler mode Q_i, where the dashed line represents the conversion efficiency with $Q_\mathrm{i} = 5 \times 10^5$. (b) Conversion efficiency of the white dashed line in (a).

[2] H. Cao and J. Wiersig, Dielectric microcavities: Model systems for wave chaos and non-Hermitian physics, Reviews of Modern Physics **87**, 61 (2015).

[3] K. Husimi, Some formal properties of the density matrix, Proceedings of the Physico-Mathematical Society of Japan. 3rd Series **22**, 264 (1940).

[4] C.-H. Yi, J. Kullig, and J. Wiersig, Pair of exceptional points in a microdisk cavity under an extremely weak deformation, Physical Review Letters **120**, 093902 (2018).

[5] C.-H. Yi, J. Kullig, C.-M. Kim, and J. Wiersig, Frequency splittings in deformed optical microdisk cavities, Physical Review A **96**, 023848 (2017).

[6] F. Fritzsch, R. Ketzmerick, and A. Bäcker, Resonance-assisted tunneling in deformed optical microdisks with a mixed phase space, Physical Review E **100**, 042219 (2019).

[7] Y. K. Chembo and N. Yu, Modal expansion approach to optical-frequency-comb generation with monolithic whispering-gallery-mode resonators, Physical Review A **82**, 033801 (2010).

[8] K. Ikeda, R. E. Saperstein, N. Alic, and Y. Fainman, Thermal and Kerr nonlinear properties of plasma-deposited silicon nitride/silicon dioxide waveguides, Optics Express **16**, 12987 (2008).

[9] Y. Sun, J. Stone, X. Lu, F. Zhou, J. Song, Z. Shi, and K. Srinivasan, Advancing on-chip Kerr optical parametric oscillation towards coherent applications covering the green gap, Light: Science & Applications **13**, 201 (2024).

[10] J. R. Stone, X. Lu, G. Moille, and K. Srinivasan, Efficient chip-based optical parametric oscillators from 590 to 1150 nm, APL Photonics **7** (2022).

[11] E. F. Perez, G. Moille, X. Lu, J. Stone, F. Zhou, and K. Srinivasan, High-performance Kerr microresonator optical parametric oscillator on a silicon chip, Nature Communications **14**, 242 (2023).

[12] J. A. Black, G. Brodnik, H. Liu, S.-P. Yu, D. R. Carlson, J. Zang, T. C. Briles, and S. B. Papp, Optical-parametric oscillation in photonic-crystal ring resonators, Optica **9**, 1183 (2022).

[13] J. R. Stone, X. Lu, G. Moille, D. Westly, T. Rahman, and K. Srinivasan, Wavelength-accurate nonlinear conversion through wavenumber selectivity in photonic crystal resonators, Nature Photonics **18**, 192 (2024).

[14] J. R. Stone, G. Moille, X. Lu, and K. Srinivasan, Conversion efficiency in Kerr-microresonator optical parametric oscillators: From three modes to many modes, Physical Review Applied **17**, 024038 (2022).

[15] F. Zhou, X. Lu, A. Rao, J. Stone, G. Moille, E. Perez, D. Westly, and K. Srinivasan, Hybrid-mode-family Kerr optical parametric oscillation for robust coherent light generation on chip, Laser & Photonics Reviews **16**, 2100582 (2022).

[16] J. Stone, D. Westly, G. Moille, and K. Srinivasan, On-chip Kerr parametric oscillation with integrated heating for enhanced frequency tuning and control, Optics Letters **49**, 3118 (2024).

[17] N. L. B. Sayson, K. E. Webb, S. Coen, M. Erkintalo, and S. G. Murdoch, Widely tunable optical parametric oscillation in a Kerr microresonator, Optics Letters **42**, 5190 (2017).

[18] N. L. B. Sayson, T. Bi, V. Ng, H. Pham, L. S. Trainor, H. G. Schwefel, S. Coen, M. Erkintalo, and S. G. Murdoch, Octave-spanning tunable parametric oscillation in crystalline Kerr microresonators, Nature Photonics **13**, 701 (2019).

[19] H. Liu, G. M. Brodnik, J. Zang, D. R. Carlson, J. A. Black, and S. B. Papp, Threshold and laser conversion in nanostructured-resonator parametric oscillators, Physical Review Letters **132**, 023801 (2024).

[20] R. J. Barbour, K. N. Dinyari, and H. Wang, A composite microcavity of diamond nanopillar and deformed silica microsphere with enhanced evanescent decay length, Optics Express **18**, 18968 (2010).

[21] M. Corato-Zanarella, X. Ji, A. Mohanty, and M. Lipson, Absorption and scattering limits of silicon nitride integrated photonics in the visible spectrum, Optics Express **32**, 5718 (2024).
